# Rapid on-line detection and grading of wooden breast myopathy in chicken fillets by near-infrared spectroscopy

**DOI:** 10.1371/journal.pone.0173384

**Published:** 2017-03-09

**Authors:** Jens Petter Wold, Eva Veiseth-Kent, Vibeke Høst, Atle Løvland

**Affiliations:** 1 Nofima AS, Norwegian Institute for Food and Fisheries Research, Muninbakken 9–13, Breivika, Tromsø, Norway; 2 Nortura SA, Lørenveien 37, Oslo, Norway; Agricultural University of Athens, GREECE

## Abstract

The main objective of this work was to develop a method for rapid and non-destructive detection and grading of wooden breast (WB) syndrome in chicken breast fillets. Near-infrared (NIR) spectroscopy was chosen as detection method, and an industrial NIR scanner was applied and tested for large scale on-line detection of the syndrome. Two approaches were evaluated for discrimination of WB fillets: 1) Linear discriminant analysis based on NIR spectra only, and 2) a regression model for protein was made based on NIR spectra and the estimated concentrations of protein were used for discrimination. A sample set of 197 fillets was used for training and calibration. A test set was recorded under industrial conditions and contained spectra from 79 fillets. The classification methods obtained 99.5–100% correct classification of the calibration set and 100% correct classification of the test set. The NIR scanner was then installed in a commercial chicken processing plant and could detect incidence rates of WB in large batches of fillets. Examples of incidence are shown for three broiler flocks where a high number of fillets (9063, 6330 and 10483) were effectively measured. Prevalence of WB of 0.1%, 6.6% and 8.5% were estimated for these flocks based on the complete sample volumes. Such an on-line system can be used to alleviate the challenges WB represents to the poultry meat industry. It enables automatic quality sorting of chicken fillets to different product categories. Manual laborious grading can be avoided. Incidences of WB from different farms and flocks can be tracked and information can be used to understand and point out main causes for WB in the chicken production. This knowledge can be used to improve the production procedures and reduce today’s extensive occurrence of WB.

## Introduction

During the last five years the muscle syndrome wooden breast (WB) has become a serious challenge to the poultry meat industry worldwide. WB is a term for abnormal muscle tissue in the chicken breast, a myopathy, which makes the breasts appear as pale, hard and out-bulging [1]. Since the appearance of this meat is unpleasant and the functional properties are impaired, severe cases of WB fillets are downgraded and used to manufacture less valuable products. With typical incidences of 5–10% of WB in markets with heavy broilers this represents significant economic losses for the poultry industry [2]. The causes for WB are still not clear, but are most likely multifactorial where an important part is related to the fast growth of modern broiler chickens [3]. With this emerging problem, it is important to find and develop methods and strategies that can alleviate the situation in the poultry industry.

Some introductory work has been done to identify non-invasive biomarkers based on mass spectroscopy for diagnostic purposes in live birds [4]. Such markers can contribute to understand the biochemical processes leading to tissue hardening. It might also be used as a trait for animal selection in breeding work. For effective handling of the problem in the poultry processing industry, however, there is a need for rapid on-line detection and grading of WB for automatic quality grading and sorting. Maybe more important, such a method would also be a valuable tool for mapping incidences of WB at a large scale and backtrack them in the production chain in order detect and remove causes of WB connected to production conditions.

There are some specific features of WB muscle tissue compared to normal muscle tissue that should enable rapid detection and grading. WB tissue has significantly higher moisture content and a significantly lower protein content. Soglia et al. [5] reported mean protein and moisture contents for WB tissue of 21.6% and 77.26%, respectively, compared to 24.65% and 73.78%, respectively for normal tissue. There were also significant higher concentrations of fat and collagen in WB tissue, but the actual differences were rather small (0.22% and 0.27%, respectively). The same researchers also measured water holding capacity by the use of low field NMR and reported that there is a significantly higher share of loosely bound water in the WB tissue, probably due to muscle fiber degeneration [5]. The characteristic pale color of the WB tissue due to the degenerative processes is not a unique marker for WB since normal fillets can also have a similar pale color. The hard texture of the WB meat, however, is notably different from normal breast meat and is today the decisive marker used in the industry for detection by manual palpation.

A good candidate method for real-time and large scale detection of WB is near-infrared spectroscopy (NIR). NIR is well suited for on-line grading and sorting of complex foods and is widely used in the food industry for determination of typically fat, water, protein and carbohydrates in products such as meat, fish, cereals and fruits [6]. Studies show that NIR spectroscopy can be used to determine fat, water and protein in chicken breast meat. Good accuracy has been obtained for samples of homogenized muscle, while NIR on intact breasts yielded poorer results with regard to average crude chemical composition [7]. The main reason for this poorer result was probably that the NIR spectra were collected in reflection mode on the surface of the breasts and did not capture internal sample heterogeneity. NIR technology has later been developed to allow improved measurements on intact heterogeneous food products by use of spectral imaging in combination with so-called interaction measurements. Interaction enables optical probing of about the upper 2 cm of the samples, which means that more representative spectral measurements can be obtained. This technical approach made it possible to design on-line NIR systems for moisture determination in very heterogeneous samples such as dried salted cod [8] and fat and color in salmon fillets [9]. An illustrative example is an application where the edible food content in live crabs is measured through the dark brown shell (carapace) [10]. NIR systems like this can also be used to determine fat content in trimmings of beef and pork [11] and it is demonstrated that this on-line method offers a good basis for real-time optimization of food processes based on automatic sorting of different qualities [12].

The objective of this work was to elucidate if an NIR imaging system can be used to detect and grade wooden breast syndrome in chicken fillets in a process line. Two approaches were tested for discrimination of wooden breast fillets: 1) Linear discriminant analysis based on NIR spectra only, and 2) a regression model for protein content was developed based on NIR spectra and the estimated concentrations of protein were used for discrimination. We also included microscopy of some selected normal and abnormal chicken breasts to verify the presence of wooden breast and to compare with NIR measurements. All NIR measurements were done in an industrial environment on chicken breasts passing on a conveyor belt at a relevant speed. The NIR system was finally tested in a real process for massive quality monitoring to log the occurrence of wooden breast in different broiler flocks.

## Materials and methods

### Chicken fillets

A total of 197 skinless breast fillets (*M*. *pectoralis major*) were taken out for analysis during three days in a commercial chicken processing facility. Average live bird weight at this plant was 1.8 kg. The fresh and chilled fillets were taken directly from the processing line approximately 3 hours after CO_2_-stunning, bleeding and slaughter of the birds. The two first days, 154 fillets were sampled with the aim of spanning all kind of normal quality variation in size, color and texture. The fillets came from different flocks and farms represented these two days. WB fillets were not sampled, but when normal fillets among the 154 had possible symptoms of WB, this was noted in the experimental log. Each fillet was scanned with the NIR system, and subsequently color and pH were measured. This procedure took about 5 minutes per fillet. The fillets were then packed in plastic bags and stored over night at 4°C, before fat, water and protein content for the whole breast was determined for 99 of these fillets (randomly selected) by low field nuclear magnetic resonance (NMR).

On day three, 15 normal, 15 moderate WB and 13 severe WB fillets were picked out of the processing line. The samples were classified by an experienced veterinarian based on visual inspection and palpation of consistency (normal, hard and very hard). Breast fillets with hard consistency and limited distribution of very hard parts were classified as moderate WB, while fillets with extensive areas of very hard consistency, were considered severe WB. WB fillets were also very pale, but no chicken breasts had significant amounts of serous fluid at the surface described from some studies in markets with higher slaughter weights. The samples were measured the same way as the first 154 samples. In addition, some muscle samples were excised for histological evaluation (see below for details). Fat, moisture and protein were determined the following day in the outer 1-cm layer of the breast fillet.

Industrial testing was performed about one year after the sampling described above. During these trials 55 normal fillets and and 24 wooden breast were again classified by a trained veterinary. Of the WB fillets, 16 were moderate and 8 were severe WB according to characteristics described above. They were scanned with the NIR scanner and no further analyses were done one these. They were used as test set for discrimination models developed based on samples from day 1–3.

### NIR measurements

The on-line NIR system was a QVision500 (TOMRA Sorting Solutions, Leuven, Belgium), an industrial hyperspectral imaging scanner designed for on-line measurement of fat in meat on conveyor belts. It was installed in the chicken processing hall at Nortura Hærland (Hærland, Norway). During the first part of the work it was equipped with a conveyor belt, but was off-line, i.e. it was not integrated in the commercial processing line. This allowed more flexibility when conducting the investigation. During the second part of the work, the scanner was installed above the actual processing line.

The NIR instrument was based on interactance measurements where the light was transmitted into the meat and then back scattered to the surface. Optical sampling depth in the chicken fillets was approximately 20 mm. Each NIR measurement took less than 1 sec. The scanner was placed 30 cm above the conveyor belt so there was no physical contact between samples and the instrument. The scanner collected hyperspectral images of 15 wavelengths between 760 and 1040 nm with a spectral resolution of 20 nm. The output per sample scan was an image of the conveyor belt with the sample. Size of the image was 60 pixels in the direction perpendicular to belt movement and 200 pixels in the direction of belt movement. Each pixel represented a spatial area of about 7 mm × 5 mm across and along the conveyor direction, respectively. The imaging capability of the used system was in this work used mainly for effective sampling, to obtain a representative mean spectrum from each fillet.

Each fillet was scanned three times with skin side of the breast facing the sensor. The replicate measurements were used to test for reproducibility.

### Determination of protein, moisture and fat

Of the 154 fillets from day 1 and 2, 99 fillets were thoroughly homogenized and two parallels of 6 g were subjected to fat and moisture determination. The average values of the parallels were used in the further work. Fat and moisture content were determined by low field proton nuclear magnetic resonance (NMR), using a Maran Ultra Resonance 0.5 tesla (Oxford Instruments, UK) equipped with a gradient probe. The method used was “The one-shot method” developed by Anvendt Teknologi AS (Harstad, Norway) [13]. Operating temperature of the magnet was 40°C and the samples were heated up to this temperature before measurement to ensure that the fat was in liquid form. The weights of all meat samples were measured and calibration was done against a reference meat sample of known weight containing 14.3% fat (SMRD 2000 Matrix Meat Reference Material, National food Administration, Uppsala, Sweden). Protein for each sample was determined as 100%—(fat% + moisture%) since water, fat and protein make up approximately 100% of the tissue weight.

For the fillets from day three (15 normal and 28 WB) only the outer 1-cm layer of muscle tissue was used for fat, moisture and protein determination. This was done to study in more detail the tissue affected by wooden breast syndrome.

### pH and color

Color was measured as L*a*b* values on the surface of the breast fillets at three locations; rostral part (thick part of fillet), middle and caudal part, with a Minolta CR-400 chromameter (Konica Minolta Sensing, Inc., Osaka, Japan). Each site of measurement covered about 1 cm^2^ of the fillet surface. The values from the three sites were averaged before further use. The instrument was calibrated once every morning with a white reference following the instrument. L* is a measure for lightness, a* expresses degree of redness (or green when values are negative), while b* expresses yellowness (or blue when values are negative). This kind of color space is commonly used for food color measurements.

pH was measured with a Knick Portamess 911 pH (Knick Elektronische Messgeräte, GmbH & Co. KG, Berlin, Germany) with the electrode InLab® Solids electrode 51343153 (Mettler Toledo, Switzerland). This was an insertion probe that was inserted about 1 cm into the rostral part of the fillet.

### Classification and calibration of NIR spectra

#### Linear discriminant analysis

From each sample scan we obtained an average intensity NIR spectrum (T). This spectrum was converted to an absorption spectrum (log10(1/T)) to make the data more linear. To remove some of the spectral variation connected to e.g. sample distance from scanner, light scattering etc., standard normal variate (SNV) was used to normalize the data [14].

A discriminant function was made based on linear discriminant analysis (LDA) [15]. Since the variables (absorption values at different wavelengths) in the NIR spectra are highly covariant, we used the score values from a partial least squares regression (PLSR) [16] of the NIR spectra on to the two classes; normal and WB. These score values are orthogonal to each other and well suited as input variables in LDA.

One NIR scan from each sample from day 1–3 were used to establish a discriminant function. This function was first validated by full cross validation to determine the optimal number of PLS factors to use in the function. It was then validated on the test set of 79 samples obtained under fully industrial conditions one year later.

### Regression model

Partial least squares regression was used to make a calibration between NIR spectra and protein and moisture concentration. Full cross validation was applied to determine the optimal number of PLS factors and to evaluate the model’s predictive ability. The prediction error was estimated by the root mean square error of cross validation (RMSECV) where *ŷ*_*i*_ is the predicted value from the cross validation, *y*_*i*_ is the reference value and *i* denotes the samples from 1 to *N*.

$$RMSECV=\sqrt{\frac{1}{N}\sum_{I=1}^{N}\left(y_{i}-\hat{y_{i}}\right)}$$

The calibration was made based on the average of the three spectra from each of the 99 referenced samples from day one and two. The average spectra were used to establish a best possible match between spectral data and chemistry. This calibration was then applied to predict protein in the samples from day three to see if it was possible to discriminate WB from normal fillets based on these values. Protein values were predicted for each of the three scans per sample. The regression model was also validated on the test set of the 79 samples obtained under industrial conditions. The main aim of the calibration model was not necessarily to obtain a best possible prediction of protein, but to obtain a good discrimination of WB from normal fillets.

One-way analysis of variance (ANOVA) was performed to analyze group differences between normal and WB. Two-way ANOVA was used to check for differences between replicate NIR scans. If the p-value was less than 0.05 the differences were considered significant.

The software The Unscrambler ver. 9.8 (CAMO Software AS, Oslo, Norway) was used for regression analysis. LDA as well as all image processing of multispectral images; sample segmentation, spectral extraction and spectral pre-processing were carried out by the use of MATLAB version 7.10 (The MathWorks Inc., Natic, MA).

### Histological evaluation

Samples for histological evaluation were taken from 10 normal, 10 moderate WB and 10 severe WB fillets. Samples for transverse sections were excised from the outer layer of the rostral region, fixed in formalin, and paraffin embedded. Sections were cut (5 μm thickness) perpendicular to the muscle fiber direction and stained using a standard haematoxylin and eosin stain. Histological evaluations were performed using a light microscope.

### On-line testing in industrial line

**Fig 1:**
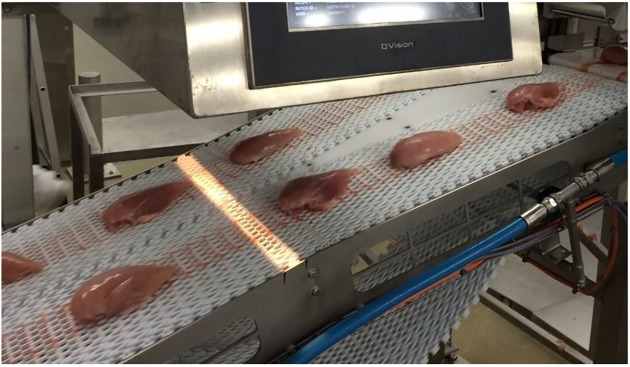
NIR system installed in production line. Illuminating line crosses the entire conveyor belt. All passing fillets were measured.

The classification model based on the protein calibration was implemented in the NIR scanner so it could detect WB fillets among chicken fillets passing on a conveyor belt. The regression model was chosen before the LDA discriminant function because it was interesting to monitor also variation in protein. The scanner was installed in a commercial processing line where the frequency of fillets was up to 3.5 per sec. Two parallel lines with fillets, separated by about 15 cm, were scanned simultaneously (Fig 1). With the scanner we collected protein values for batches of fillets belonging to different broiler flocks, and the values were automatically written to file. We collected data for 66 flocks from different farms and typical number of fillets per batch was between 6.000–18.000. About 8.500 fillets could typically be scanned per hour depending on the frequency of fillets on the line. The position of scanning in the line was after the point were the most severe cases of WB were removed manually by the workers. That means that the cases of WB that were scanned in these trials were mostly of moderate condition. Nortura SA gave permission to this field study.

## Results and discussion

### Fat, water and protein, color and pH

Table 1 summarizes the approximate composition (fat, water and protein) of the fillets. Note that measurements on normal and WB fillets on day three were done on the outer 1 cm of the breast to study the region mostly affected by WB. This is also the layer that will have the greatest impact on the NIR spectra. Water and protein levels for normal fillets day 3 were similar to corresponding values for the 99 fillets sampled day 1 and 2. Fat content was significantly different by 0.25%-points. This means that approximate composition of the outer 1-cm tissue layer in normal fillets was the same as for the whole fillet as measured days 1 and 2. The WB fillets had significantly lower protein content compared to the normal fillets, mean differences of 4.6% and 5.1% for moderate and severe WB, respectively. Similar differences (3.8% and 4.3%, respectively) were found for moisture. Soglia et al. [5] found the same systematic changes in approximate composition between normal and WB tissue, but they found smaller differences, probably because they measured the whole fillets and not the outer 1-cm layer in the most affected part of the fillet, as in this study.

**Table 1:** Approximate chemical composition, color and pH in normal breast muscle and wooden breast muscle.

	Normal day 1&2	Normal day 3	Moderate WB	Severe WB
	Whole fillet	Upper 1 cm	Upper 1 cm	Upper 1 cm
	(n = 99)	(n = 15)	(n = 15)	(n = 13)
Moisture %	74.9 ± 0.86	75.3 ± 0.66	79.1 ± 1.49°*	79.6 ± 1.49°*
Protein %	23.5 ± 0.89	23.5 ± 0.64	18.9 ± 1.22°*	18.4 ± 1.47°*
Fat %	1.6 ± 0.62	1.25 ± 0.50°	1.8 ± 0.53	2.0 ± 0.67°*
	(n = 154)			
L*	56.10 ± 3.70	52.7 ± 2.68°	60.3 ± 1.7	59.8 ± 2.3°*
a*	2.97 ± 1.32	2.46 ± 0.62	2.34 ± 0.91	4.56 ± 2.9*
b*	7.41 ± 2.92	5.19 ± 1.22	8.84 ± 1.48*	10.52 ±1.85°*
pH	5.99 ± 0.12	6.3 ± 0.10°	6.3 ± 0.16°	6.3 ± 0.09°

Average value ± standard deviation. Values in shaded fields are from outer 1-cm layer of breast fillets.

* indicates significant different from group mean value of normal fillets day 3.

° indicates significant different from group mean value of normal fillets day 1&2.

There was a clear tendency that the WB fillets had high L*-values, meaning that they were pale. They also had high b* values indicating yellowness. Although significant differences in L*, a* and b* between severe WB and normal fillets, the color variation could not be used to distinguish WB from normal, since many normal fillets were also quite pale.

There were no differences in pH between the groups on day 3, but average pH day 3 was higher than average pH day 1 and 2. We are not sure of the reason for this. Fillets day 3 were sampled during a shorter time span compared to fillets day 1 and 2, which were collected over two full days. The difference might therefore be ascribed to a flock effect.

### Histological characterization

**Fig 2:**
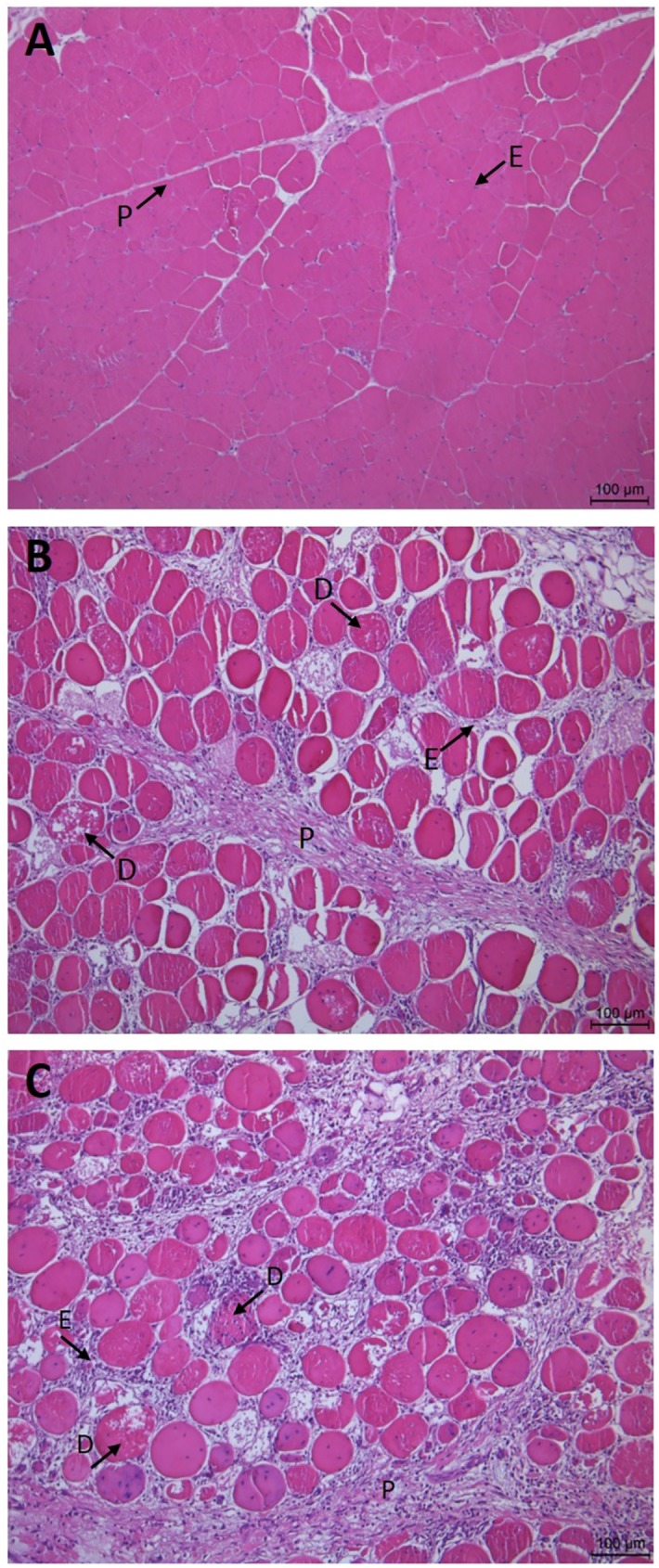
Morphologic structures in chicken breast muscle. Normal (A), moderate (B) and severe WB (C) (M. pectoralis major). D = degenerating fibers; E = endomysium; P = perimysium.

In order to verify the presence of wooden breast in the selected fillets on day 3, histological evaluations of 10 normal and 20 WB fillets were performed. Fig 2 shows representative light microscopy images of cross sections from normal (a), moderate (b) and severe WB fillets (c). In the normal fillets, the classical structure of skeletal muscle can be seen, with tightly packed polygonal muscle fibers of relatively even diameter. Moreover, each muscle fiber is surrounded by a thin layer of endomysium, and bundles of muscle fibers are surrounded by a slightly thicker layer of perimysium. In the WB fillets, on the contrary, various signs of myopathy could be seen. Specifically, the muscle fibers in the WB fillets are rounded and appear to be more variable in size. Many of the muscle fibers also show signs of degeneration and infiltration of inflammatory cells, and these features increase in incidence from moderate to severe WB fillets. In addition, the endo- and perimysium layers have thickened. These findings are in consistence with earlier reports on myopathy and wooden breast in the pectoralis major muscle of broilers [1, 17–18], and thus confirms the WB phenotypes in the selected fillets.

### NIR spectra

**Fig 3:**
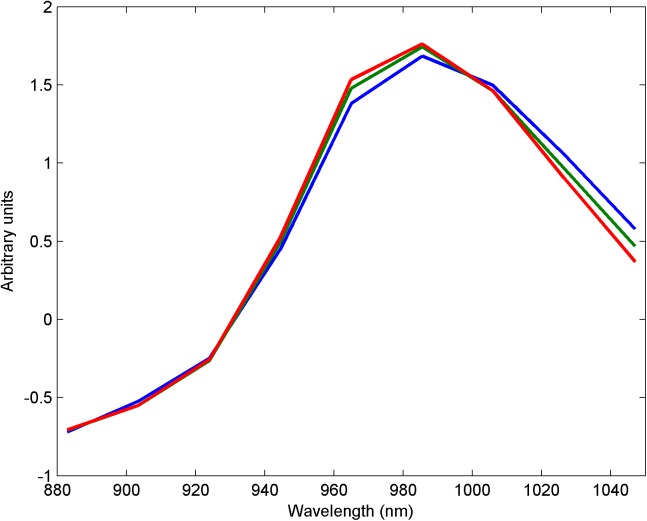
Typical NIR spectra from chicken fillets. Normal fillet (blue), moderate WB (green) and severe WB (red) fillets. Spectra were measured on samples A, B and C, respectively, shown in Fig 2.

Fig 3 shows SNV corrected NIR spectra from a normal and two WB fillets of different severity, the same samples as shown in Fig 2. The main absorption peak for moisture in this spectral region is at about 980 nm. A close look at the spectra reveals a systematic shift of the peak towards shorter wavelengths with the severity of WB. This shift was quite prominent and constituted the main variation in the spectra from the chicken fillets. The spectral changes can most likely be attributed to two phenomena: 1) Protein has an absorption peak at about 1020 nm (not directly discernable in the spectra). A lower protein concentration and a corresponding increase in moisture could contribute to the observed spectral shift. This corresponds with the systematic differences in water and protein in WB tissue as described above. 2) It is well known that a shift in the water peak at 980 nm does occur when the water molecules are more or less bound to other molecules [19]. More loosely bound water creates a shift towards shorter wavelengths. It has been reported that there is significantly more loosely bound water in WB compared to normal fillets [5] and it is likely that it will create a shift as observed. The same characteristic shift in the absorption peak at 980 nm has been proposed as a method to detect human breast cancer due to a larger share of less bound water compared to normal breast tissue [20]. Thus, both the effects are probably in action and emphasize the differences between normal and WB muscle tissue.

### Classification

#### Linear discriminant analysis

**Fig 4:**
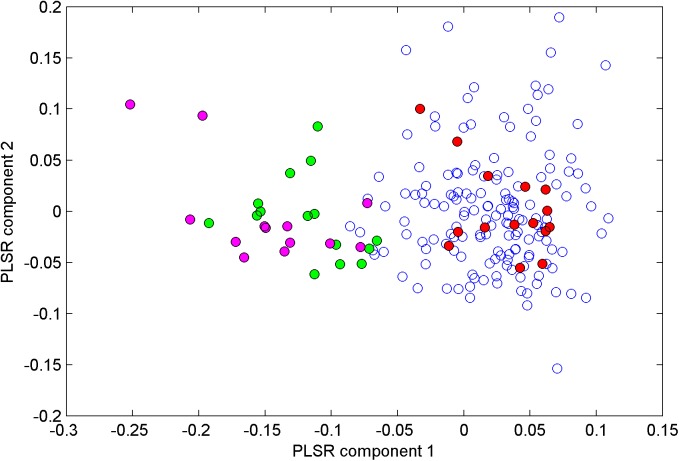
PLSR score plot for NIR spectra. Score values for PLSR components 1 and 2 for normal fillets from days 1 and 2 (blue), normal fillets day 3 (red), moderate WB (green) and severe WB (magenta).

PLSR score values for all samples measured day 1–3 are shown in Fig 4. Component 1 expressed 54% of the variation in the NIR spectra, separating normal and WB fillets quite well. The score values of component 1 correlated closely with the protein content of the samples (R = 0.90). A cross validated model based on 3 PLSR components gave 99.5% correct classification of the 197 samples. All 28 WB fillets were correctly classified and only one normal fillet was classified as WB. This is a good result taking into consideration that there was a gradual change in muscle tissue from normal to WB. In a system with gradual change it will always be some misclassification close to the decision line. The discriminant function did also work very well on the test set recorded under industrial conditions one year later. All samples were correctly classified, indicating that the NIR spectra contained systematic and clear differences between the groups of fillets.

#### Regression analysis

Table 2 summarizes cross validated calibration results for water and protein in chicken fillets based on the NIR spectra from normal fillets collected day one and two. The rather low correlations obtained were reasonable since the range in e.g. protein was short (20.5–25.3%). Prediction errors (RMSECV), however, were quite low and indicated that protein and moisture could be estimated with an accuracy of approximately ± 0.57% and ± 0.58%, respectively. This result is comparable with what was obtained by the use of reflectance NIR on intact chicken breasts [7].

**Table 2:** Calibration results for chemical constituents in whole chicken fillets based on NIR imaging scanner.

	# LV^*a*^	R^*b*^	RMSECV^*c*^ (%)
Protein	4	0.76	0.57
Moisture	6	0.67	0.58

^*a*^# LV—number of latent variables in the PLS regression model.

^*b*^R—Correlation coefficient.

^*c*^RMSECV—Root mean square error of cross validation.

**Fig 5:**
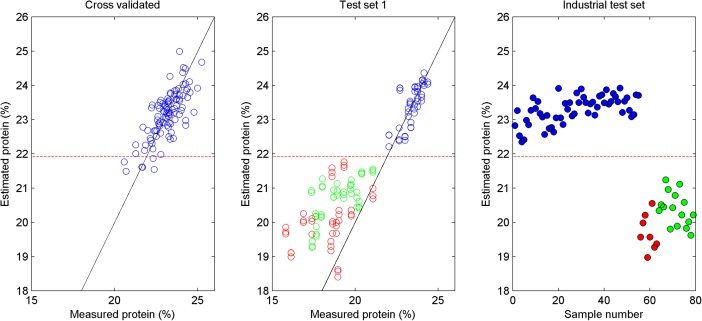
NIR estimated protein in chicken fillets. *a*) Cross validated predicted versus measured protein in normal chicken fillets. *b*) Estimated protein in samples from day 3. Normal (blue), moderate WB (green) and severe WB (red). *c*) Estimated protein in test samples. Normal (blue), moderate WB (green), severe WB (red). The dashed red line at 21.9% indicates the decision line between normal and WB.

The regression model obtained for protein (Fig 5*A*) was used to estimate protein content in the samples from day 3 (Fig 5*B*). The normal fillets got estimated protein values above about 22% and lied along the target regression line. All WB samples got protein estimates below 22%. Estimated protein values for many of the WB fillets were slightly higher than the measured protein concentration in these fillets. There are different reasons for this: 1) Protein was measured in the upper 1 cm layer of the breast, while the NIR system did probably measure deeper than 1 cm, and the spectra were affected by more normal tissue deeper than 1 cm with higher protein concentration. 2) The calibration model was not calibrated with samples of such low protein values, meaning that the model was extrapolating and larger deviations from the true protein content could be expected. 3) Spectral shifts due to loosely bound water were not included in the regression model.

Since the regression model did not include WB samples, we can more strictly say that the WB fillets were discriminated by outlier detection. This approach is in line with established methodology within statistical process control: The normal variation of samples or process conditions are modeled, and the models are used to detect deviations from the normal [21]. We did try to include all samples from day 1–3 in the same regression model (normal and WB), but this approach did not work well with regard to classification. The main point is that the protein model did clearly separate between normal and WB fillets. A linear discriminant analysis of the predicted protein values for the groups of normal and WB resulted in an optimal decision limit at 21.9%. The model for moisture could also separate between normal and WB fillets, but not as clear as the protein model.

The model did also work very well on the 79 test samples from the industrial trial (Fig 5*C*). Again normal fillets got protein values above 22% and WB below. Note that there were a few samples in the calibration set (Fig 5*A*) with protein content less than 21.9%. Four out of these samples were listed as “possible WB” in the experimental log during the experimental work.

In both test sets (Fig 5*B* and 5*C*) there was a tendency that the severe WB had lower estimated protein levels compared to the moderate WB group, but there were no significant difference between the two groups.

Fig 5B shows that the replicate NIR scans resulted in very similar protein predictions. There was no significant difference between the replicate protein estimates, indicating good reproducibility of the NIR measurements.

The results show that both LDA and the calibration model for protein are very well suited for classification of WB from normal fillets. The very high percentage of correct classification obtained in this study is rather optimistic. As noted above, there is a gradual change in muscle properties from normal to WB. This means that in the region of the decision line there will be miss-classifications in a grey zone. In a practical industrial setting, different decision limits can be chosen according to needs and experience. Two limits can for instance be used to separate the fillets into high, medium and poor quality.

An advantage with the classification model based on protein values is that it gives additional information. Monitoring the protein content can be of interest in the poultry industry. This method is also intuitively easy to understand for operators of such a system. Adjusting the decision line in a LDA model is more complex and less intuitive.

An important reason for the good results is connected to the measurement mode of the NIR spectra. It seems that for severe cases of WB a thicker part of the breast muscle is affected by myopathy. An efficient grading based on NIR will therefore require that the light penetrates rather deep into the muscle, as it did in this study. Reflectance measurements at the surface might work to separate WB from normal muscle, but would most likely be a less accurate method. Degree of severity can also be defined by how much of the fillet surface that is affected. Some fillets are affected on mainly the thick rostral part while on other fillets, the complete breast fillet is affected. This difference can be captured by the NIR system used in this study since the entire fillet is measured, not only a limited region.

### Industrial on-line measurement trials

**Fig 6:**
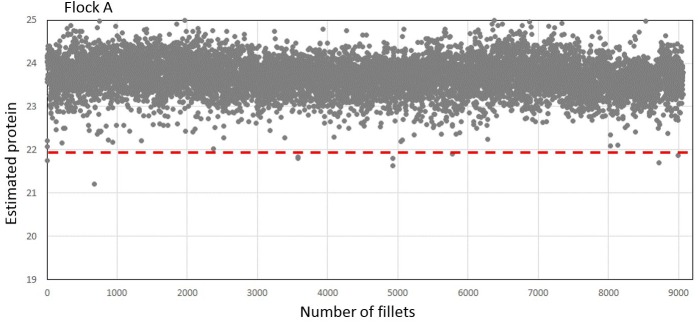
NIR estimated protein in flock A. Red horizontal line indicates the chosen threshold of 21.9% protein.

Fig 6 shows estimated protein values for all fillets from one flock of birds from farm A (approximately 9063 fillets). Only 8 samples were below the chosen threshold of 21.9%. This was a flock with very low incidence of fillets with protein estimates typical for WB.

**Fig 7:**
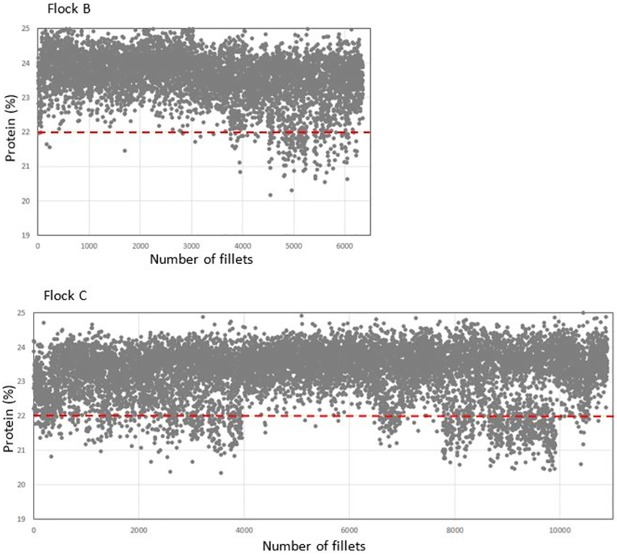
NIR estimated protein in flock B (upper panel) and C.

Fig 7 shows similar recordings from two other farms, flock B (6330 fillets) and C (10483 fillets). In those cases the incidence of breast fillets with low protein estimates, indicating WB, was considerable higher. The x-axis in the plots count the numbers of fillets and do also indicate sequence of measurement. The patterns indicate that there were higher incidences of WB in certain parts of the batch, maybe coinciding with certain houses at the farms or other production factors.

**Fig 8:**
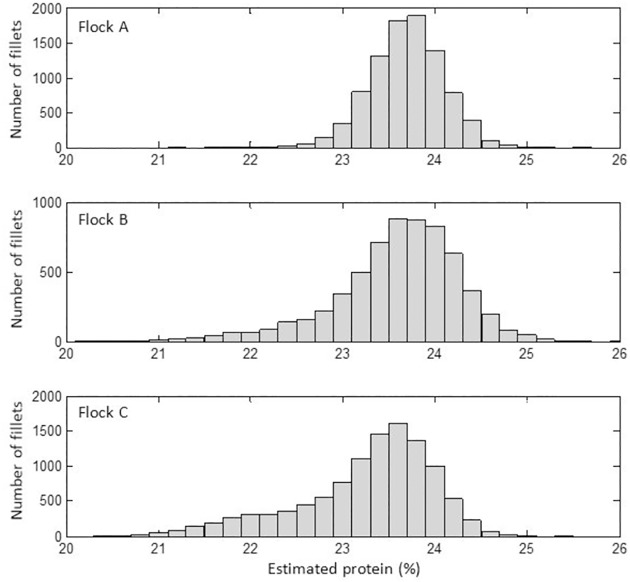
Histograms showing distribution of estimated protein in the broiler flocks A, B and C.

Fig 8 illustrates the protein distribution in fillets from the three farms as histograms. Most values were centered around 24%. Farm A had a more or less Gaussian shape around 24%, while farm B and C had bigger tails towards lower protein values. Farm B and C had prevalences of 6.6% and 8.5% of protein estimates below 21.9%, respectively, which were the highest incidences of WB in the 66 flocks that were screened in this period.

## Conclusion

The results shows that on-line interactance NIR is a well working, practical and useful tool for detection and grading of WB syndrome. Low protein content is clearly characteristic for WB, which is shown in this study and also by others [5]. At this point it is not clear if the main variation in the NIR spectra are attributed to differences in protein and water, or to the amount of less bound water. The two effects would probably co-vary, but it should be further studied in order to understand the system in the best possible way.

An on-line system as presented here can be used to alleviate the challenges wooden breast represent to the poultry meat industry. It can be used for two important tasks: 1) It is possible to automatically sort chicken fillets of different quality to different product categories. Manual grading and removal of WB can then be avoided and replaced by a much more rapid and objective system. 2) It is possible to track incidences of WB in detail from different farms and use this crucial information to understand and point out main causes for WB in chicken production. This knowledge can be used to improve the production procedures and reduce today’s extensive occurrence of WB.

## Supporting information

S1 File Spectroscopic and reference data for all samples. (XLSX)

S2 File Description of supplementary data. (DOCX)
