# Supplementary material for: Rapid on-line detection and grading of wooden breast myopathy in chicken fillets by near-infrared spectroscopy
Source: PLoS One. 2017 Mar 9;12(3):e0173384. doi: 10.1371/journal.pone.0173384 (PMC5344484; doi:10.1371/journal.pone.0173384)
Supplement: S2 File — (DOCX) [file pone.0173384.s002.docx]

**Description of supplementary data**

The data is organized in an Excel data sheet.

SNV corrected NIR spectra are provided for all samples (for day 1, 2 and 3 and for test set recorded under industrial conditions). The spectra are the average spectra of three scans. The three scans per sample were not significantly different.

Color (L*, a* and b*) and pH are provided for all samples day 1,2,and 3

Chemical data (fat, water and protein) is provided for 99 samples recorded day 1 and 2 nad for all samples recorded day 3.

The data columns are organized as:

1. Sample number
2. Sampling time (day 1,2,3 or industrial sampling one year later)
3. Class (normal or Wooden breast) Wooden breast are characterized in two classes: 1 – moderate, 2 – severe.
4. pH measured at sampling time
5. Fat%
6. Protein%
7. Water%
8. Mean L*
9. Mean a*
10. Mean b*

K-Y Wavelengths in NIR spectra.
